# Rate-disabled versus accuracy-disabled subtypes of dyslexia: A longitudinal study from preschool to grade 1

**DOI:** 10.1007/s11881-026-00379-w

**Published:** 2026-07-08

**Authors:** Dana Gott, Ravit Cohen-Mimran, David L. Share

**Affiliations:** 1 Department of Learning Disabilities, University of Haifa, Haifa, Israel; 2 Edmond J. Safra Brain Research Center for the Study of Learning Disabilities, Haifa, Israel; 3 Department of Communication Sciences and Disorders, University of Haifa, Haifa, Israel

**Keywords:** Dyslexia, Subtypes, RAN, Phonological awareness, Morphological awareness, Reading accuracy, Reading rate

## Abstract

This longitudinal study investigated subtypes of dyslexia based on the distinction between word reading accuracy and word reading rate. Building on prior cross-sectional research, we inquired whether the cognitive profiles of these subtypes can be identified before children learn to read. A large sample of Hebrew-speaking children were assessed on phonological awareness (PA), morphological awareness (MA), and rapid automatized naming (RAN) in preschool and then followed into first grade to evaluate their reading accuracy and rate. In preschool, findings revealed two distinct subgroups: one with selective deficits in PA and MA but intact RAN (PA + MA-disabled) and another with impaired RAN but preserved PA and MA (RAN-only disabled). In first grade, as predicted, the PA + MA-disabled group demonstrated significantly lower reading accuracy compared to both the RAN-only disabled and control groups. The RAN-only subgroup exhibited slow reading but intact accuracy. However, contrary to predictions, the PA + MA-disabled group also exhibited slower reading rates. These results suggest that at the beginning of reading development, low levels of reading accuracy limit reading rate. Our study (i) supports accuracy-rate subtyping of dyslexia, (ii) reinforces the role of both PA and MA in achieving early word reading accuracy, and (iii) highlights the existence of a highly specific RAN-rate dyslexia subtype. These findings have significant implications for early diagnosis and intervention as well as the definition of dyslexia.

Literacy skills are essential in modern life, not only for academic learning, but also for occupational success and overall quality of life, and yet a considerable number of children, even in literacy-rich cultures, struggle to acquire reading expertise (Norton & Wolf, 2012; Snow et al., 1998; Snowling & Melby-Lervåg, 2016). Many studies have sought to pinpoint the antecedents of reading problems. Today, there is a wide consensus that the cognitive basis of many specific (and non-specific) reading difficulties is a phonological deficit (see e.g., Brady et al., 2011; Jorm & Share, 1983; Rayner et al., 2001; Shankweiler & Liberman, 1989; Share, 1995; Snowling, 1998; Stanovich, 2000; Vellutino et al., 2004; Wagner & Torgesen, 1987). The phonological deficit hypothesis, however, does not presume to explain all the variance in reading disabilities (Nation & Snowling, 2004; Share & Stanovich, 1995). It is now clear that at least some aspects of phonological processing (such as PA) may be relatively unimpaired in some disabled readers (O’Brien et al., 2012; Shechter-Lerner et al., in press; Wolf & Bowers, 1999) particularly so in relatively transparent orthographies. These considerations highlight the possibility of multiple sources of reading disability (Pennington et al., 2012; Ramus et al., 2003). The notion of heterogeneity among dyslexics has understandably motivated attempts to demarcate subtypes of reading disability (see, e.g., Boder, 1971,1973; Fletcher et al., 1997; Lyon et al., 1982; Mattis et al., 1975; Morris et al., 1998; Stanovich & Siegel, 1994) but many typologies have been grounded more in clinical intuitions and/or untested theoretical preconceptions than in a well-developed and rigorously tested theory of reading. A notable exception is the subtyping approach inspired by the influential Coltheart/Baron dual-route model of word reading (Coltheart et al., 1993, 2001) which has attracted considerable interest (as well as controversy) among Anglophone reading disability researchers (see, e.g., Bailey et al., 2004; Castles & Coltheart, 1993; Harm & Seidenberg, 1999; Jackson & Coltheart, 2001; Kim, 2021; Manis et al., 1996; Milne et al., 2003; Stanovich et al., 1997).

According to the dual-route approach (Castles & Coltheart, 1993) there are two distinct subtypes of dyslexia; one displaying a selective impairment in the lexical reading route (“surface” dyslexia) and the other displaying a specific impairment in the non-lexical or sub-lexical route (“phonological” dyslexia). Because it is a product of the classic dual-route model, this subtyping scheme, however, is primarily focused on the challenges of (oral) reading accuracy in the highly irregular English orthography. It may be less relevant to other more transparent orthographies, such as German or Spanish, in which print-sound correspondences are straightforward and “exception” words are rare or non-existent (Hutzler et al., 2004; Shany & Share, 2011; Share, 2008; Ziegler & Goswami, 2005). In most regular phoneme-based orthographies (i.e., alphabets and (Semitic) abjads), children typically reach ceiling levels of accuracy by the end of the first grade and even dyslexics read quite accurately. Rather, it is speed and fluency that is the discriminating factor in individual and developmental differences. These observations suggest that subtyping schemes which take into account reading speed and not just reading accuracy may offer a more widely applicable alternative to the conventional surface/phonological typology (Shany & Share, 2011).

Lovett (1984, 1987) was the first to propose a subtyping framework based on the distinction between reading rate and reading accuracy. She classified a clinical sample (ages 8–13) into accuracy-disabled and rate-disabled subgroups. To be subtyped as “rate-disabled,” a child had to score “close to, at, or above grade level” on accuracy and at least 1.5 years below grade level on reading speed. To be subtyped as “accuracy-disabled,” a child had to score poorly on word recognition accuracy, but because no rate criterion was designated for this group, they were also impaired in reading rate, and, in fact, were slower readers than the rate-disabled group. Thus, the accuracy-disabled group were, overall, more severely impaired readers, or “doubly-disabled,” that is rate- *and* accuracy-disabled, whereas the rate-disabled were only “singly-disabled”. Thus, these data are vulnerable to an alternative “severity” account which argues that speed and accuracy are two correlated measures tapping a single common underlying construct; the rate-disabled subgroup are simply less severely disabled on this common dimension than the accuracy-and-rate disabled subgroup. By this account, a unidimensional taxonomy can still be maintained simply by distinguishing different levels of severity on this continuum. Lovett did not explore the possibility of true double dissociation by attempting to identify an accuracy-only disabled group who read at normal rates.

A subsequent subtyping study by Leinonen et al. (2001) also identified two subgroups with selective deficits in either accuracy or rate. Among a sample of Finnish dyslexic adults, a subgroup of accuracy-disabled (“hasty”) readers was identified. A second subgroup of rate-disabled (“hesitant”) readers were slow readers but made few reading errors. These findings suggest a clear dissociation between accuracy and rate, but the fact that the reading speed of the hasty group and accuracy of the hesitant group were lower than normal levels, makes it difficult to establish a true or pure rate-accuracy differentiation in the sense of intact performance on one dimension in the presence of impaired performance on the other dimension.

Another relevant, and influential subtyping approach is the Double Deficit Hypothesis (DDH) of Wolf and Bowers (1999). This hypothesis reflects a different theoretical approach to the present accuracy/rate classification, because it takes as its point of departure the RAN-PA dissociations found either in conventionally defined dyslexics (Wolf & Bowers, 1999) or in the general population of preschoolers (Ozernov-Palchik et al., 2017). According to the DDH, difficulties in PA and serial naming speed (RAN) deficits represent two distinct causes of reading disability, with slow RAN associated with slow reading, whereas poor PA is characterized by inaccurate reading and spelling. Ozernov-Palchik et al. (2017) examined the longitudinal stability of these subtypes in a large sample of US kindergarten children followed from preschool to Grade 2. On the basis of preschool measures of PA, RAN, letter knowledge and verbal short-term memory, latent profile analysis revealed six different profiles including PA-risk, RAN-risk, and double deficit risk. The PA-only and RAN-only profiles were found to be stable from the very beginning of reading acquisition through to the end of first grade (the second year of formal reading instruction in the US). Steacy et al. (2014) also found evidence of “moderate” stability in these groups over time, with a higher probability of stability between first and second grade. It should be noted that both these longitudinal studies did not independently define reading-disabled groups in terms of accuracy-disabled or rate-disabled but looked at later accuracy and fluency (a measure that combines accuracy and speed) of their RAN-only and PA-only subgroups.

Shany and colleagues (Shany & Share, 2011; Shany et al., 2023), reported evidence of true double dissociation between subtypes of dyslexia defined on the basis of either reading accuracy and/or reading rate in cross-sectional studies of Hebrew-speaking and Arabic-speaking fourth graders. It is important to note that Shany and colleagues measured *pure* word reading rate (i.e., the number of words read correctly or incorrectly in a given time interval), not fluency which is conventionally defined as the number of words read *correctly* in a given time interval. Analysis of a large nationally representative sample of fourth grade children revealed three distinct subtypes: accuracy-disabled with normal reading speed; rate-disabled with intact reading accuracy; and a doubly-disabled group with performance below normal levels on both accuracy and rate measures. Each of these groups was found to have similar prevalence, each comprising approximately one third of the dyslexic subsample and 10% of the population sample. Furthermore, the two single-deficit subgroups also showed distinct cognitive and linguistic profiles. The rate-specific subgroup exhibited slow RAN, while the accuracy-specific subgroup was characterized by selective deficits in PA and MA. Whereas deficits in both PA and MA were consistently found in our accuracy-only disabled subgroups in both our published studies (and also in several additional unpublished cross-sectional studies now in preparation), findings for vocabulary and syntax were not consistent. Although these profiles suggest a mild form of general language impairment (DLD), our data repeatedly indicate that PA and MA are the two most reliable markers of the accuracy-disabled profile. It is noteworthy that the inclusion of morphology, alongside phonology, as a defining feature of the accuracy-disabled subtype aligns with contemporary research indicating (i) a central role for morphology in reading development and reading difficulties across languages (Berthiaume et al., 2018; Rastle, 2023; Levesque et al., 2021; Kirby & Bowers, 2018; Hasenäcker et al., 2021) and (ii) a growing consensus that the roots of many reading difficulties including dyslexia can be found in broader and earlier linguistic deficiencies and not just phonology (Adlof & Hogan, 2018; Catts, 1996; Catts & Kamhi, 2005; Scarborough et al., 2005; Snowling & Hulme, 2021; Snowling & Melby-Lervåg, 2016).

Both Shany’s published studies cited above were cross-sectional, focusing on children who had already been taught to read. This raises the question of whether the distinct cognitive-linguistic profiles identified in these studies are causes or consequences of reading disability. Particularly noteworthy is the accuracy-disabled subtype, which exhibited notable deficiencies in PA and MA. Limited exposure to written materials might have resulted in missed opportunities to enhance their linguistic abilities (Share & Silva, 1987; Stanovich, 1986). Thus, it is important to explore the predictive value of these cognitive and linguistic profiles, particularly PA and MA, *before* formal reading instruction commences.

The present longitudinal study examines these domains prior to the onset of reading instruction. In Israel, unlike the US, Kindergarten is the third and final pre-school year and is entirely separate, physically and institutionally from elementary school which begins in Grade 1 when children are taught to read. Thus, our longitudinal investigation provides a relatively “clean” test of the predictive association between preschool PA, MA, RAN, and later reading profiles, one uncontaminated by reciprocal relationships between these abilities and reading. We ask whether there are significant numbers of children in Kindergarten who only have RAN deficits but normal PA and MA, as well as children with the reverse pattern ‒ normal RAN but deficient PA/MA deficits? Do these children later develop selective problems with reading rate and reading accuracy once they come to school and are taught to read? Specifically, do children with early difficulties in language (PA and MA) but not RAN become inaccurate but not slow readers, whereas the RAN-only subgroup become slow but accurate readers?

## Method

### Participants

The study sample included 1,146 Hebrew-speaking children from 128 kindergartens in and around the Haifa region and other settlements in the north of Israel. These children were later followed into their elementary schools, typically located in the same neighborhood. The kindergartens included in this study were located in diverse residential locations including urban and rural settlements (kibbutzim and moshavim) encompassing a wide range of socio-economic backgrounds. Socioeconomic status (SES) for each participant was determined using a comprehensive national ranking system (1 = lowest SES; 10 = highest) provided by the Israeli Central Bureau of Statistics (CBS). Each kindergarten was assigned an SES score (1–10) based on the socioeconomic ranking of the locality or neighborhood in which it was located, as defined by the CBS classification of residential areas across Israel.

Both non-religious and religious kindergartens were included in the study, but ultra-orthodox kindergartens which follow a different educational curriculum, as well as special education kindergartens, were excluded. No child was excluded due to developmental or learning/attentional disorders or a non-Hebrew-speaking home background. This inclusive approach aligned with the study’s overarching aim of tracking the development of early literacy, numeracy, and cognitive skills in a representative sample of Hebrew-speaking children. By focusing on a broad spectrum of learning disabilities including dyslexia, dysgraphia, dyscalculia, ADHD, and DLD - we purposefully included a wide range of abilities, ensuring that children at-risk or experiencing difficulties were fully represented.

The cohort was longitudinally monitored from kindergarten to elementary school. It is important to note that the testing process was abruptly halted in March 2020 due to the outbreak of the COVID-19 pandemic, which resulted in widespread school closures. From the original kindergarten sample 30 children were retained for an additional year in kindergarten, 6 children had transferred to special education programs, 10 families had relocated to different parts of the country, five parents chose to withdraw their children from the study, and 415 children were unable to complete the full set of tests before school shutdown. Also, 157 children who made two or more errors on at least one of the RAN tests were also removed from the sample, although supplementary analyses confirmed that the exclusion of this subsample did not alter the pattern of results reported below. A total of 340 children completed the entire test battery in kindergarten and first grade.

### Sub-group selection

The subgroups were formed as in our previous cross-sectional studies (Shany & Share, 2011; Shany et al., 2023) using a 25th percentile low achievement cut-off. The RAN-only disabled group (*n* = 46) scored below the 25th percentile on the RAN composite but higher than the 35th percentile on the PA + MA composite. Conversely, the PA + MA-only disabled group (*n* = 54) exhibited a selective deficit on the PA + MA composite scoring below the 25th percentile but above the 35th percentile on the RAN composite.^1^ The control group (*n* = 138) was designated as those children scoring above the 35th percentile but below the 65th percentile on both the RAN and the PA + MA composites.^2^

Formal reading instruction in Israel begins only in Grade 1 and, almost universally, adheres to a decoding-emphasis approach in which children are taught the symbol-sound correspondences of the basic phonic building blocks of pointed Hebrew. These are the CV units (*tserufim*) consisting of a consonant letter with vowel sign beneath as well as isolated (vowelless) consonant letters (see Shechter & Share, 2025, for more details). In Kindergarten, children are taught to break spoken words into their constituent sounds (syllables, CVs and Cs) and are also taught the names (and sometimes sounds) of the letters but are not taught to read (i.e., decode) words.

Ethical approval was granted by the Ministry of Education - Office of The Chief Scientist (permit #9667) and the Institutional Review Board at the XXXX Faculty of Education of the University of XXXXX. Parents gave their informed written consent to allow their child to participate in the data collection.

### Procedure

All participants were individually tested on the measures described below as part of a wide-ranging battery of tests related to language, arithmetic, executive functions, reading, and writing. These measures were administered in two phases: preschool measures (including PA, MA, and RAN) were administered at the end of the kindergarten (preschool) year (May-June), and reading abilities were assessed in mid-1st Grade (January-March). All testing took place in a quiet setting in the kindergarten/school and without the presence of the teacher. The duration of each session ranged from 30 to 40 min, depending on the child’s pace, and the language tests were presented orally without any written material. All examiners received instruction and training in each test administration by the research team. All testing personnel had a background in education, psychology, or speech pathology.

### Materials

Most measures were either created by the authors or modified from existing clinical or research tools. This adaptation process involved two stages. Firstly, the number of original items was reduced to accommodate the wider battery’s time constraints, and items were chosen to be suitable for kindergarten children. All measures were then tested on a pilot sample of 50 children. Based on criteria such as item difficulty, internal consistency, and test-retest reliability, items were added or removed. The test description reports the number of items that were retained in each task. See Table 1 for means, standard deviations, as well as internal consistency and test-retest reliability data.

**Table 1:** Means, standard deviations, ranges, and reliabilities for kindergarten phonological and morphological measures, RAN time, early literacy measures, and mid-grade 1 reading measures (accuracy and rate)

Measure	Range	M (SD)	Reliability
Non-verbal general ability (Raven)	1–18	11.1 (2.98)	
PA			
Initial consonant isolation in CVC words	1–10	3.5 (3.79)	0.84
Initial consonant isolation in CCVC words	1–10	4.6 (3.84)	0.89
Final consonant isolation in CVC words	1-12–	4.1 (3.96)	0.87
MA			
Resultative Adjective Derivation	1–10	5.3 (2.54)	0.77
Verb Derivation	1–8	4.5 (1.91)	0.84 Test-retest = 0.79
Noun Pluralization	1–15	10.6 (2.69)	0.76 Test-retest = 0.63
Pseudoverb inflection	1–10	5.0 (2.35)	0.71 Test-retest = 0.50
RAN			
RAN colors (time in seconds)		76.0 (25.88)	Test-retest=0.86
RAN objects (time in seconds)		73.7 (19.83)	Test-retest=0.87
Early (Kinderarten) literacy			
Letter naming	1–10	6.6 (2.61)	0.86 Test-retest = 0.91
Letter recognition	1–10	8.2 (2.21)	0.82 Test-retest = 0.82
Word choice	1–12	5.0 (2.78)	0.78
Word Reading Accuracy (%) Grade 1			
CV Reading	1–23	89.1 (13.96)	0.91 Test-retest = 0.58
Word reading /ɑ/	1–60	84.2 (18.75)	0.81
Word reading all vowel signs	1–40	49.7 (26.44)	0.85
Pseudoword reading /ɑ/	1–30	76.9 (24.54)	0.80
Word Reading Rate Grade 1			
CV reading (time in seconds)		33.0 (18.96)	
Word reading /ɑ/ (total words read in 60 s)		24.0 (8.61)	0.81
Word reading all vowel signs (total words read in 60 s)		18.7 (7.55)	0.83
Pseudoword reading /ɑ/ (total words read in 60 s)		15.1 (5.55)	0.78

#### Phonological (Consonant) Awareness

PA was assessed with three tasks. All tasks consisted of common Hebrew syllable structures (CVC, CCVC) taken from a corpus of 50 words commonly used by toddlers aged between 1;4 and 3;3. These words were selected from a longitudinal database created by Ruth Berman (Berman, 1990). Each task included 10–12 items. Half of the items began with stop consonants, and half with continuants. Test items were preceded by a demonstration item and three practice items with corrective feedback.

The following tasks were included:

#### Initial consonant isolation in CVC words

The children were asked to repeat a spoken target word and identify the initial consonant. The task consisted of 10 target items (e.g. say the word /mɪts/ ‘juice’, at the beginning of the word /mɪts/ we hear…? /m/). One demonstration item and three practice items with feedback were presented before the task began. A correct answer earned 1 point.

### Initial Consonant Isolation in CCVC Words

The children were asked to repeat a spoken target word and identify the initial consonant. This task also consisted of 10 target items (e.g. say the word /dvɑʃ/ ‘honey’, at the beginning of the word /dvɑʃ/ we hear…? /d/). One demonstration item and three practice items with feedback were presented before the task began. A correct answer was awarded 1 point.

### Final consonant isolation in CVC words

The children were asked to repeat a spoken target word and identify its final consonant. This task contained 12 items (e.g. say the word /dɑɡ/ ‘fish’. at the end of the word /dɑɡ/ we hear…? /ɡ/). One demonstration item and three practice items with feedback were presented before the task began. A correct answer received 1 point.

For each of the PA tests, mean scores were transformed into Z scores, then averaged to construct a single composite measure of PA. Principal Components Analysis indicated that the first principal component explained 68% of the variance, with high and very similar factor loadings around the 0.80 mark for each component.

#### Morphological awareness

The MA tasks require the child to identify a morphological relationship in a pair of words (verb/adjective, noun/verb, noun/noun) presented in a demonstration sentence. The child was then required to apply this relationship to other pairs of words and solve the morphological analogy by generating the appropriate word. For example, children were asked to transform one part of speech (such as a verb) to an adjective through morphological changes (see specific examples for each task below). It should be noted that the derivation test items offer multiple semantically acceptable options to complete the sentences, but only the correct derivation was considered correct.

#### Resultative adjective derivation

This task, adapted from Yegev (2001), required the children to derive 10 adjectives from spoken verbs. For instance, children were asked to complete sentences such as: “/XɑTXu/ (they cut) the apple. (Root letters appear in uppercase lettering.) Now the apple is __________ /XɑTuX/, ‘cut’)”; 2. “/SiDʁu/ (they arranged) the books. Now the books are __________ (/meSuDɑʁim/, arranged)”. Thus, the child is required to derive the adjective from the given verb at the beginning of the sentence. It is crucial to note that in highly inflected languages such as Hebrew, unlike English in which verbal and adjectival (and often nominal) forms have the identical surface form (cut/cut, arranged/arranged), derived forms in Hebrew differ markedly. For example, /XaTXu/ (חתכו) is a verb in the KAL pattern (one of the seven verbal patterns), conjugated in masculine plural past tense. In contrast, /XaTuX/ (חתוך) is an adjective derived from the same root but in the CaCuC pattern (one of many adjectival patterns). During practice, a correct response, which included a derivation of the correct adjectival form from the verb, was acknowledged with positive reinforcement (“good”), followed by the tester repeating the completed sentence. If an incorrect answer (or no answer) was given, the tester provided the correct derivation and repeated the entire sentence. A score of one point was given for each correct response.

#### Verb derivation

This task (adapted from Novogrodsky & Kreiser (2015)) required children to generate eight verbal patterns based on spoken noun roots. The task began with a demonstration item (“What do we do with the /TSɛVɑʕ / (color)? With the /TSɛVɑʕ/ /TSoVʕɪm/”) followed by a practice item: “What do we do with the /mɛXɑDɛD/ (pencil sharpener)? With the pencil sharpener /məXɑDəDɪm/”. A correct answer that included the appropriate verb derivation received positive feedback from the tester, followed by a repetition of the completed sentence. If the child provided an incorrect or no answer, the tester provided the correct derivation and repeated the entire sentence. One point was given for each correct derivation.

#### Noun pluralization

This task (adapted from Lavie (2006) and Yegev (2001) required the child to generate 15 plural forms of singular nouns, each accompanied by a picture of the given object. The test contains five regular forms (three masculine and two feminine nouns) and eight irregular forms (six masculine and two feminine nouns) of the plural inflectional suffix. The target nouns consisted of an equal number of regular and irregular suffixes, with some containing unaltered stems, while others involve stem-internal changes. For example, the masculine noun /kɪʁ/ ‘wall’ takes the feminine plural marker ‘ot’ to create /kɪʁot/ ‘walls’. Before the test items were given, the children received two practice items with feedback. A correct answer was followed by repetition of the completed sentence with the correct inflection. If an incorrect answer (or no answer) was given, the tester provided the correct plural form followed by the completed sentence. Each correct answer earned one point.

#### Pseudoverb inflection

A short version of the SHALAZ test developed by Cohen-Mimran et al. (2018). Participants were introduced to a novel verb containing a phonologically legal but non-existent root in the Hebrew language and were required to complete 10 sentences using that verb in the correct inflection according to the morpho-syntactic rules of the Hebrew language. For example: “אתמול הוא שָלַז; אתמול היא ____ (שָלְזָה)” (‘Yesterday he _______ /ʃɑLɑZ/; Yesterday she ______? /ʃɑLZɑ/). Each correct answer scored 1 point.

Raw scores for each of the four MA tests were transformed into Z scores, then averaged to construct a single composite measure of MA. Principal Components Analysis showed that the first principal component explained 60% of the variance.

Finally, a composite PA + MA measure combining phonological and morphological awareness was created by averaging the separate composite Z scores for the PA/MA composite measure.

#### Rapid automatized naming (RAN)

Because substantial numbers of Kindergarten children did not know many of the names of the Hebrew letters or digits, we used non-alphanumeric RAN – colors and objects – to assess rapid automatized naming (Denckla & Rudel, 1976).

### Rapid automatized naming (RAN) of Colors

Children were asked to name five rows of five color patches (black, blue, green, red and yellow) as quickly as possible. Prior to the test, the tester verified that each child knew the names of the selected colors.

#### Rapid automatized naming (RAN) of Objects

Children were asked to name five rows of five objects (lion, ball, chair, flower and clock) as quickly as possible. Once again, prior to the test, the tester verified that each child knew the names of the selected objects.

Children who committed two or more errors on one or both of the RAN tests (*n* = 157) were excluded from the study.^3^ A single composite RAN measure was calculated based on the averaged performance of the two RAN times.

#### Kindergarten early literacy measures

For the purpose of assessing early literacy knowledge in kindergarten, three tests were developed.

#### Letter naming

The children were asked to name 10 letters of the Hebrew alphabet, including word-final letters, randomly presented. Test items were preceded by two practice items (one regular letter and one final letter) with demonstration and feedback.

#### Letter recognition

The children were asked to identify a named target letter among a set of four alternatives. The test consisted of 10 letters, including word-final letters. Target letters were presented in a randomized order and preceded by two practice items (one regular letter and one final letter) with demonstration and feedback.

#### Word choice

Adapted from Van der Kooy-Hofland et al., 2012. This Hebrew version of the Dutch test contained 12 items each consisting of a row of four printed words. Participants were asked to point to the word that matched a spoken word. The three distractors matched either the target spelling’s first letter, the first two letters, or no letters at all. For example, for the target word כלב (‘dog’), the distractors were (2-letters) כלא (‘prison’), (one letter) “כעס” (‘angry’) and (no shared letters) עין (‘eye’). The correct answer was awarded three points, choice of the distractor with two identical letters was awarded two points, the distractor with the single matching letter was awarded one point, with no points for the distractor that was entirely different from the target word.

### Grade 1 Reading Measures

First grade reading was assessed by tasks measuring speed and accuracy of decoding CV (consonant-vowel) combinations, real words and pseudowords. At this point of the school year children had been taught all the consonant letters and the three most common vowel signs, which each denote the default vowel /ɑ/ which accounts for around half of all the vowels in Hebrew. In accordance with this curriculum, we developed four reading tasks. First, children were asked to read a list of all 23 consonant letters with the vowel diacritic /a/ while reading errors were recorded by the examiner. These CV units are the basic building blocks of the Hebrew abjad and are often taught as integral units referred to as *tserufim* (see Authors, 2025). Total reading time for the complete list was recorded. The next three tests were adapted from the TOWRE word and pseudoword reading tests (Schiff et al., 2012; Katzir et al., 2012). One reading list consisted of 60 fully pointed words containing all the consonants but only the /ɑ/ vowel signs. The second list consisted of 30 pseudowords also featuring only the vowel /ɑ/. This list of pseudowords was based on the real words to ensure that all items preserved Hebrew’s morphological patterning. The third and final list included 30 real words containing the complete set of Hebrew vowel signs. All word lists were organized in columns using identical font and size. Except for the first CV reading task in which total time reading was measured, the word and pseudoword lists were allotted a 60 s time limit. Reading errors were recorded in all lists. All words in the reading tests were fully pointed (voweled). The word lists were presented on a printed page in columns and all the words were displayed in the same font and size. The order of the tasks was fixed, beginning with the CV list, followed by real words with only the default /ɑ/ vowel, then the pseudoword list, and concluding with the real words with all the vowels.

For each task, two scores were calculated: One for rate – the total number of words read (correctly or incorrectly) in 60 s, and one for accuracy - the percentage of words correctly read out of the total number of words the subject attempted to read. It is crucial to reiterate that the present measure of reading rate was a pure measure of reading speed unlike conventional measures of reading fluency which are typically the number of words correctly read in a fixed time interval.

Principal Components Analysis was used to combine the three word and pseudoword reading measures. The first principal component accounted for 82% of the variance with high and very similar factor loadings (between 0.88 and 0.93) for each of the rate variables. For reading accuracy, the first principal component accounted for 76% of the variance, again with high and very similar factor loadings (between 0.78 and 0.94) for each of the individual accuracy variables.

#### Cognitive measures

##### Non-Verbal General Ability (Raven’s Matrices)

The colored version of Raven’s Standard Progressive Matrices was used to measure nonverbal ability (Raven et al., 1998). Following an explicit demonstration example, participants were asked to select the missing part of a geometric pattern from a set of alternatives. A total of 18 items (only even-numbered items) were included in this assessment. Raw scores were converted to a standardized score with a mean of 100 and standard deviation of 15. Children identified with low general ability who scored below 70 were excluded from the sample.

## Results

Prior to conducting our main analyses, we performed several preliminary checks to ensure the validity of our group selection procedures. First, we sought to confirm that the groups were comparable in terms of background variables: age, gender, socioeconomic status, and general ability. We then compared the three groups on the selection variables – PA, MA, and RAN (see Table 2). 

**Table 2:** Means and standard deviations (in parentheses) for definitional and background measures for three groups (PA+MA-only, RAN-only, and a typically achieving control group

	Group 1 PA + MA-only disabled (*N* = 54)	Group 2 RAN-only disabled (*N* = 46)	Group 3 Control group (*N* = 138)	Statistics
Age	5.7 (0.47)	5.5 (0.31)	5.6 (0.38)	*F(3,302) =* 1.53* n.s.*
Gender	57.4% boys	34.8% boys	40.6% boys	*F*(3,336) = 2.17 *n.s.*
Kindergarten SES (scale 1–10)	5.5	6.0	6.4	*F*(3,336) = 2.35 *n.s*.
General ability score (Raven)	10.4	11.0	11.6	*F*(3,336) = 2.28 *n.s.*
PA + MA Z score	-0.8^b^ (0.28)	0.3 ^a^ (0.40)	0.3^a^ (0.40)	*F*(3,336) = 98.61*** 1 < 2 = 3
RAN times (in seconds.)	64.3^a^ (6.66)	100.8^b^ (24.42)	62.9 ^a^ (7.31)	*F*(3,336) = 105.36*** 2 > 1 = 3

*p* < 0.001***, *p* < 0.01**, *p* < 0.05*

Different superscript letters (^a, b^) indicate significant group differences

Analysis of background variables revealed no significant differences between groups in age, gender distribution, kindergarten SES, or general ability scores. On the other hand, there was a clear and true double dissociation on the selection measures. The PA + MA-only disabled group showed significantly impaired PA + MA Z scores compared to both RAN-only disabled and control groups, but intact performance on the RAN tests. Conversely, the RAN-only disabled group demonstrated significantly slower RAN times compared to PA + MA-only disabled and control groups alongside intact PA + MA performance.

### Sub-group performance on kindergarten early literacy measures

To address the primary research question whether a true double dissociation, characterized by selective deficits in RAN versus PA and MA, exists before children learn to read, we first established the prevalence of these distinct cognitive profiles. Table 3 presents the descriptive statistics for kindergarten morphological and phonological measures (percentages).

**Table 3:** Means and standard deviations (in parentheses) for early literacy measures for three subgroups in kindergarten (PA + MA-only, RAN-only, and typical readers)

	Group 1 PA + MA-only disabled (*N* = 54)	Group 2 RAN-only disabled (*N* = 46)	Group 3 Control group (*N* = 138)	Statistics
Letter name retrieval	5.7^b^ (2.53)	6.9^a^ (2.34)	7.5^a^ (2.28)	F(3,336) = 10.25*** 1 < 2 = 3
Letter recognition	7.5^b^ (2.23)	8.3^a^ (1.96)	8.7^a^ (2.02)	*F*(3,336) = 5.65*** 1 < 2 = 3
Word recognition	3.9^b^ (2.23)	5.4^a^ (1.96)	6.6^a^ (2.02)	*F*(3,336) = 16.91*** 1 < 2 < 3

*p* < 0.001***, *p* < 0.01**, *p* < 0.05*

Different superscript letters (^a, b^) indicate significant group differences

Different superscript letters (^a, b^) indicate significant group differences.

Table 3 shows that the PA + MA-only disabled group performed significantly lower than the RAN-only disabled and control groups on all three early literacy measures. The RAN-only disabled group, in contrast, did not differ significantly from the control group on any measure. It is important to note that all three early-literacy measures assessed accuracy alone and were not timed or speeded measures. This result converges with our previous cross-sectional work showing that, as a group, the rate-only disabled subgroup (defined on the basis of Grade 4 reading rate alone) evinced a highly selective deficit in RAN but no difficulties with reading *accuracy*. The present findings extend our earlier work by showing that a group defined solely on the basis of slow preschool RAN (but intact PA + MA), that is, with the same cognitive-linguistic profiles as our fourth-grade rate-disabled readers demonstrated unimpaired levels of accuracy on preschool early literacy measures. Also affirming and extending our early cross-sectional findings, the PA + MA-only subgroup consistently demonstrated poorer performance on all three early literacy (accuracy) measures.

### Sub-group performance on grade 1 reading accuracy

This section addresses the question of whether the selective cognitive profiles identified in Kindergarten ultimately lead to a dissociation in reading outcomes (accuracy vs. rate). We specifically examined whether the children with PA+ MA deficits developed into inaccurate readers, and whether the RAN-only subgroup developed into slow but accurate readers. Performance of the three groups in first grade in terms of reading accuracy is presented in Table 4.

**Table 4:** Means (in percent correct) and standard deviations (in parentheses) for Grade 1 reading accuracy in three groups (PA + MA-only disabled, RAN-only disabled and control group)

	Group 1 PA + MA-only disabled (*N* = 54)	Group 2 RAN-only disabled (*N* = 46)	Group 3 Control group (*N* = 138)	Statistics
CV reading	87.7 (9.28)	89.0 (15.92)	90.2 (13.19)	*F*(3,336) < 1.0* n.s.*
Word reading /ɑ/)	73.1^b^(22.95)	88.3^a^ (13.40)	87.4^a^ (15.61)	*F*(3,336) = 8.99*** 1 < 2 = 3
Word reading all vowel signs	36.8^b^ (23.29)	55.3^a^ (27.61)	56.5^a^ (24.99)	*F*(3,336) = 9.58*** 1 < 2 = 3
Pseudoword reading /a/	64.7^b^ (25.83)	82.3^a^ (22.17)	83.2^a^ (21.74)	*F*(3,336) = 9.52*** 1 < 2 = 3

*p* < 0.001***, *p* < 0.01**, *p* < 0.05*

Different superscript letters (^a, b^) indicate significant group differences

As predicted, the group with both phonological and morphological weaknesses but not RAN – the PA + MA subgroup – scored significantly below the control group and the RAN-only group on all three word reading accuracy measures. Notably, these differences were large and not merely statistically significant. The RAN-only subgroup’s scores were almost identical to the control group means, again confirming that, as a group, their reading accuracy is intact as seen in their Kindergarten early literacy profiles above and in our previous cross-sectional work.

Interestingly, all groups showed a high degree of mastery (around 90%) of the CV units – the basic phonic building blocks of Hebrew decoding. We return to this in the discussion.

### Sub-group performance on grade 1 reading rate

First grade reading rate for the three subgroups is presented in Table 5.

**Table 5:** Means and standard deviations (in parentheses) on measures of Grade 1 reading rate for three subgroups (PA + MA-only disabled, RAN-only disabled, and the control group)

	Group 1 PA + MA-only disabled (*N* = 54)	Group 2 RAN-only disabled (*N* = 46)	Group 3 Control group (*N* = 138)	Statistics
CV reading (time in seconds)	38.1^b^ (21.11)	39.3^b^ (28.83)	28.27^a^ (13.13)	*F*(3,336) = 6.05** 3 < 1 = 2
Word reading /ɑ/ (total number of words read in 60 s.)	21.2^b^ (7.50)	22.4^b^ (8.15)	26.8^a^ (8.48)	*F*(3,336) = 8.85* 3 < 1 = 2
Word reading all vowel signs (total number of words read in 60 s.)	17.4^b^ (8.18)	16.6^b^ (7.44)	20.7^a^ (7.66)	*F*(3,336) = 5.07** 3 < 1 = 2
Pseudoword reading /ɑ/ (total words read in 60 s.)	13.8^b^ (5.54)	14.22^b^ (6.45)	16.5^a^ (5.30)	*F*(3,336) = 5.48** 3 < 1 = 2

*p* < 0.001***, *p* < 0.01**, *p* < 0.05*

Different superscript letters (^a^,^b^) indicate significant group differences

Consistent with predictions, the RAN-only subgroup showed significantly lower performance than the control group on all four measures confirming a selective deficit in reading rate (but not reading accuracy).

However, contrary to predictions, the PA + MA subgroup was also significantly slower than the control group on each of the four measures indicating that this subgroup, at least in mid-Grade 1, are both inaccurate *and* slow.

### Supplementary RAN analyses

To further validate our findings and allay potential concerns regarding the validity of the RAN measure due to naming errors in young children, we conducted a series of supplementary analyses. We first note that RAN was designed as a measure of retrieval speed in populations for whom this material (letters, digits, colors) is highly “overlearned”. However, in our sample, we found that many children had not mastered all the color and object names. The errors made by these children often involved skipping items or a whole line which affected the recorded time and calls into question the validity of this measure as a reflection of name retrieval speed.

Consistent with these observations, we found that the RAN times for the high-error subgroup of 157 – those who made two or more errors – were actually *faster* (69.4 (sec) vs. 74.9, *t*(535) = 3.16 (*p*=0.002) than the remainder of the sample who committed either zero or one error. We therefore consider this exclusion criterion justified in order to maintain the integrity and validity of the core rate variable. Nonetheless, to allay any lingering doubts, we reanalyzed our data combining the “trimmed” sample we reported above (*n* = 340) with the 157 discarded children to check if the pattern of results changed. When we compared the two sets of results on the Kindergarten and grade 1 measures (*including* versus *excluding* the 157 error-prone subsample) we found a very similar pattern of results which we report below. The reanalysis of reading data with and without the 157 error-prone children is shown below.

On the Kindergarten accuracy measures, two of the original three measures revealed the same outcome – the PA + MA subgroup but not the RAN-disabled subgroup differed significantly from the control group Table 6.

**Table 6:** Means and standard deviations (in parentheses) for early literacy measures for three subgroups in kindergarten (PA + MA-only, RAN-only, and typical readers)

Total sample *N* = 497				
	Group 1 PA + MA-only disabled (*N* = 77)	Group 2 RAN-only disabled (*N* = 69)	Group 3 Control Group (*N* = 247)	Statistics
Letter name retrieval	5.6^b^ (2.63)	6.8^a^ (2.21)	6.2^ab^ (2.75)	*F*(3,452) = 6.39*** 1 = 3, 1 < 2, 2 = 3
Letter recognition	7.6 (2.27)	8.1 (2.19)	8.5 (2.53)	*F*(3,452) = 1.24, *n.s.*
Word recognition	4.3^b^ (1.92)	5.0^a^ (2.51)	5.4^a^ (2.83)	*F*(3,452) = 4.02** 1 < 2 = 3

*p* < 0.001***, *p* < 0.01**, *p* < 0.05*

Different superscript letters (^a, b^) indicate significant group differences

As regards the Grade 1 accuracy measures, a similar pattern emerged. With the exception of the word reading (all vowel signs) task which fell marginally short of overall significance (but with the familiar pattern of results), only the PA + MA subgroup showed impaired reading accuracy Table 7.

**Table 7:** Means (in percent correct) and standard deviations (in parentheses) for Grade 1 reading accuracy in three groups (PA + MA-only disabled, RAN-only disabled and control group)

Total sample *N* = 497				
	Group 1 PA + MA-only disabled (*N* = 77)	Group 2 RAN-only disabled (*N* = 69)	Group 3 Control Group (*N* = 247)	Statistics
CV reading	87.5 (12.49)	88.8 (14.42)	82.4 (26.03)	*F*(2, 452) = 2.09 *n.s*
Word reading /ɑ/	59.9^b^ (34.18)	80.1^a^ (25.80)	74.4^a^ (32.51)	*F*(2, 452) = 8.25*** 1 < 2 = 3
Word reading all vowel signs	33.8 (27.63)	44.0 (36.21)	43.2 (31.03)	*F*(2, 452) = 2.20 *n.s*
Pseudoword reading /ɑ/	60.7^b^ (31.00)	79.2^a^ (25.52)	75.8^a^ (25.74)	*F*(2, 452) = 9.03*** 1 < 2 = 3

*p* < 0.001***, *p* < 0.01**, *p* < 0.05*

Different superscript letters (^a^, ^b^) indicate significant group differences

**Table 8:** Means and standard deviations (in parentheses) on measures of Grade 1 reading rate for three subgroups (PA + MA-only disabled, RAN-only disabled, and the control group)

Total sample *N* = 497				
	Group 1 PA + MA-only disabled (*N* = 77)	Group 2 RAN-only disabled (*N* = 69)	Group 3 Control Group (*N* = 247)	Statistics
CV reading (time in seconds)	37.6^b^ (21.41)	39.0^b^ (28.83)	28.3^a^ (13.00)	*F*(3,452) = 5.865** 1 = 2 > 3
Word reading /ɑ/ (total number of words read in 60 s.)	21.3^b^ (9.01)	22.2^b^ (9.78)	28.8^a^ (10.56)	*F*(3,452) = 23.468*** 1 = 2 < 3
Word reading all vowel signs (total number of words read in 60 s.)	17.8^b^ (7.71)	17.5^b^ (7.78)	21.2^a^ (8.22)	*F*(3,452) = 13.695** 1 = 2 < 3
Pseudoword reading /ɑ/ (total words read in 60 s.)	14.8^b^ (5.08)	14.5^b^ (6.31)	17.2^a^ (5.31)	*F*(3,452) = 11.479** 1 = 2 < 3

*p* < 0.001***, *p* < 0.01**, *p* < 0.05*

Different superscript letters (^a^, ^b^) indicate significant group differences

Turning to the results for reading rate, the data revealed the identical pattern of significantly slower performance of both the PA + MA and the RAN-only subgroups on all 4 of the reading rate measures. We conclude that the exclusion of the 157 participants did not alter our findings in any substantial way Table 8.

## Discussion

This study set out to examine the preschool precursors of accuracy-rate dyslexia subtypes within a prospective longitudinal framework. To date, prior studies have validated this framework cross-sectionally with fourth graders in Hebrew (Shany & Share, 2011) and in Arabic (Shany et al., 2023). The present study sought to shed light on the roots of these subtypes before children learn to read. We first established that the unique cognitive-linguistic profiles observed among selectively rate-disabled or accuracy-disabled readers in Grade 4, are already present among pre-literate children. One subgroup was characterized by selective deficits in PA + MA but with intact RAN; a second subgroup exhibited the opposite pattern – impaired RAN but preserved PA + MA. This replicates the cognitive-linguistic double dissociation observed in our fourth graders.

We then examined the early literacy profiles of these two subgroups focusing on early (preschool) letter knowledge and word recognition. Once again, we found clear dissociations: the PA + MA-only disabled group performed significantly below both the control group and the RAN-only disabled group across all three measures. In contrast, the RAN-only disabled group did not differ significantly from the control group on any of these measures. It is important to keep in mind that all these early literacy measures only assessed accuracy, not speed. Our first-grade measures, on the other hand, evaluated both dimensions of word reading.

Replicating our early literacy findings in preschool (Kindergarten in Israel), the PA + MA-disabled subgroup, once again, demonstrated significantly lower first grade reading accuracy across all three word reading tasks (real words with /ɑ/ vowels, real words with all vowels, and pseudowords). The RAN-only disabled group, by contrast, showed normal levels of reading accuracy that were indistinguishable from the control group. One notable finding was the high level of accuracy (around 90%) achieved by all groups in reading the basic CV units - the fundamental building blocks of Hebrew decoding (see Share, 2017). This suggests that even children with PA + MA deficits can master these basic (sub-syllabic) phonic elements in a highly transparent orthography when explicitly taught. However, their difficulties become apparent when faced with more complex orthographic patterns. These findings provide evidence that selective deficits in early reading accuracy have their roots in broader language difficulties (both phonological and morphological) that precede formal reading instruction. This corroborates a growing body of evidence that the source of many reading difficulties including dyslexia can be found in broader and earlier linguistic deficiencies and not just phonology (Adlof & Hogan, 2018; Catts, 1996; Catts & Kamhi, 2005; Price et al., 2022; Scarborough et al., 2005; Snowling & Hulme, 2021; Snowling & Melby-Lervåg, 2016).

Our study, however, puts a new twist on this language-based explanation of dyslexia. Both our cross-sectional and longitudinal data indicate that the language-based account only applies to *accuracy*-based reading disability, not to rate-based disabilities. Language deficits are indeed present in a majority of dyslexics including not only the selectively accuracy-disabled subgroup but also the accuracy-plus-rate (“doubly disabled”) subgroup which we did not include in this study, but which we repeatedly find to make up around one third of all our dyslexics. That is, the two accuracy-disabled subgroups (singly and doubly disabled) together count for around two thirds of all our disabled readers. This would explain why conventional “whole-group” studies of dyslexia consistently find statistically significant group-wise differences on language measures while overlooking a subgroup of dyslexics without language-based impairments. Our data repeatedly demonstrate that rate-only-disabled dyslexics are not impaired in the domain of spoken language. And this implies that interventions that focus on PA and/or MA are unlikely to benefit this second subgroup. For this subtype, interventions that specifically target reading rate and fluency are needed (e.g., Breznitz et al.’s RAP 2013; Frederiksen et al.’s SPEED 1985; Repeated Reading, Lee & Yoon, 2017; Shany & Biemiller, 1995; and Wolf et al.’s RAVE-O 2009). For doubly-disabled accuracy-plus-rate readers, both language-based *and* rate/fluency-based interventions will be needed.

Unlike the clear distinctions observed between these subgroups regarding reading accuracy, the pattern of results for reading rate was somewhat more complex than anticipated. As already noted, the RAN-only disabled group read significantly more slowly than controls across all measures. This RAN-and-rate-disabled subtype appears to represent a particularly pure or specific reading difficulty. However, contrary to predictions, the PA + MA group also demonstrated slower reading speeds that matched the RAN-only disabled group. The same finding was reported in a parallel study conducted by our research group among Arabic-speaking children: a PA + MA-only disabled subgroup (identified in preschool) was later found to be inaccurate and slow readers in mid-Grade 1 (Jabbour-Danial et al., 2024). These findings may reflect the fact that in the early stages of reading development, accuracy and speed are highly interdependent. It appears that, unlike the clear distinction found in Shany and her colleagues’ fourth grade studies – at which point reading skills are more firmly established – these beginning stages of reading acquisition remain primarily focused on acquiring basic decoding skills. Tracking these groups into later grades will be necessary to determine whether these PA + MA-disabled readers eventually develop normal reading speeds once they achieve higher levels of accuracy. We base this prediction on the fact that this subgroup has intact RAN, at least in preschool.

### Theoretical and educational implications

Our study provides evidence that the roots of rate-accuracy dyslexia subtypes can be found in distinct cognitive-linguistic dissociations existing prior to formal reading instruction. This bodes well for differential early identification and prevention that is tailored to specific subtype profiles. Recent reviews of intervention research make it clear that intervention research still treats dyslexics and other struggling readers as a single homogeneous group (Donegan & Wanzek, 2021; Al Otaiba et al., 2023; Stevens et al., 2021). The practical implications of the findings presented here are clear: different subtypes require different interventions.

Struggling readers who are slow but accurate readers as well as those who are both slow and inaccurate both require interventions with an explicit speed/automaticity component. Dyslexics who are both slow *and* inaccurate will also need instruction that targets their decoding difficulties and language skills – not only PA but also MA. Our results also go beyond the classic phonological deficit hypothesis of dyslexia by highlighting the role of MA alongside PA. This aligns with growing evidence for the importance of broader language skills, particularly morphology, in reading development and reading difficulties.

Reinforcing our previous cross-sectional findings, the present longitudinal investigation demonstrates that the separation of accuracy and speed has both theoretical validity and far-reaching practical implications. Indeed, both the DSM-5 and the IDA 2025 definitions of dyslexia acknowledge that there are two distinct and separable dimensions of word reading – “*inaccurate or slow and effortful word reading*” (APA, 2013), “*difficulties in word reading and/or spelling that involve accuracy*,* speed*,* or both*…” IDA 2025. It is particularly noteworthy that the earlier 2002 IDA definition of dyslexia which referred to word reading accuracy and *fluency* has now been revised to refer to accuracy and *speed.* The distinction between word reading *fluency* and word reading *speed* – raw speed – is fundamental to the definition of dyslexia and the accuracy-rate typology. This point is worth belaboring. Contrary to our approach, popular measures of word reading fluency or efficiency such as Brus and Voeten’s Dutch One Minute Test (1972) and Wagner, Rashotte, and Torgesen’s (English) TOWRE-2 (2012) typically ask participants to read aloud a list of isolated words (often graded for difficulty), stopping the reader after a fixed time limit such as one minute or 45 s. Reading performance is scored as the number of words read *correctly* in the time limit (Words-*correct*-Per-Minute or WcPM) which combines accuracy and rate into a single composite measure. This composite measure, however, confounds two distinct and dissociable sources of reading difficulty which not only have distinctive reading and cognitive-linguistic profiles but, crucially, very different instructional/intervention implications.

To measure *pure* speed or, as we prefer to label it, *rate*^4^, we ask participants to read aloud a list of isolated words from beginning to end while recording accuracy and time to complete the list. We do not stop the reader after a fixed period of time. Because all participants read the identical set of words, this provides a pure measure of accuracy (independent of rate) as well as a pure measure of rate (independent of accuracy), unlike time-limited fluency/efficiency tests which discontinue the test after a fixed period of time. In time-limited fluency/efficiency tests, the measurement of accuracy is complicated by the fact that most children end up reading a different subset of words. This problem is particularly acute if the word list is graded for difficulty. When all children read the identical set of words, the speed measure is also pure in the sense that it is based on the same set of words and independent of the accuracy with which the reader reads those words. Our measure of reading speed, therefore, differs fundamentally from the popular Words *correct* Per Minute (WcPM) metric. Consider the following WcPM scores for a hypothetical 50-word list time-limited to 60 seconds.

**Table:** 

Child	Pure rate (WPM)	Errors	Accuracy%	WcorrectPM
Typical	50	5	90%	45
A	30	0	100%	30
B	50	20	60%	30

It is clear that both child A and child B have reading difficulties; it is equally clear that they are struggling for very different reasons. Whereas A is a slow but accurate reader, B’s difficulty is with accuracy not speed. Their identical fluency/efficiency (WcPM) scores mask the fact that these two readers have very different reading profiles. We have shown in two independent, nationally representative samples of Hebrew-speaking and Arabic-speaking fourth graders, that selectively disabled readers like A and B really exist, and in significant numbers (Shany & Share, 2011; Shany et al., 2023). Furthermore, these distinct reading profiles also exhibit distinctive cognitive-linguistic profiles that call for different interventions. The present longitudinal study further demonstrates that these cognitive-linguistic profiles can be observed before children learn to read thereby permitting early identification and the promise of tailored intervention.

Although we have repeatedly observed pure accuracy-disabled and pure rate-disabled subtypes among elementary-school children from the end of Grade 2 up (see Shecter et al., 2026), the present study did not reveal a “pure” accuracy-disabled subgroup in Grade 1. We suspect that the slow and inaccurate profile of the PA + MA subgroup may be the result of the low levels of reading accuracy among our novice (mid-Grade 1) readers that may be limiting the pace of reading. Our PA + MA subgroup averaged 73% correct reading “easy” words that contained only the simple and ubiquitous /ɑ/ vowel, and only 37% reading words with all the vowel signs. This *low-accuracy- constrains-speed* interpretation is consistent with the “accuracy-before-speed” view of early word reading development proposed by both Lovett (1984a, 1984b, 1987) in English and by Juul et al. (2014) in Danish. Juul et al. (2014) cite a study by Nielsen et al., (1992) showing that low accuracy puts a cap on reading speed producing a banana-shaped distribution of accuracy plotted against speed (see Fig. 1, p. 1097, Juul et al., 2014). In their longitudinal study of Copenhagen children, Juul et al. found that speed of word reading develops only after a certain level of accuracy has been achieved (70% in Danish). According to this account, pure accuracy-disabled subgroups only emerge in late Grade 2 once children have mastered the code in a transparent orthography such as pointed Hebrew. As already noted, we predict that these accuracy-disabled beginning readers whose reading is also slow, will attain typical reading speeds once accuracy reaches higher levels. This account predicts that in a deep orthography such as English, pure accuracy-disabled readers would not be seen until later, only after the learning-to-read stage which typically lasts around three years.

Finally, we acknowledge that the data reported here derive from a non-European (Semitic) language written in a highly transparent orthography taught via code-emphasis methods. We do not yet have data on the prevalence of our accuracy-rate subtypes in other languages and writing systems. It seems reasonable, however, to predict that in opaque orthographies such as English, Danish, and French, accuracy-disabled profiles may emerge later eventually becoming more common simply because the challenges of attaining word reading accuracy are greater. Future investigations will need to consider how orthographic transparency, morphological complexity and instructional approach influence the relative prevalence of rate- and accuracy-disabled subtypes.

## Data Availability

Data will be made available to interested parties on reasonable written request to DLS.

## Footnotes

1 Of the 54 PA + MA-only subgroup, 18 were found to be below the 25th percentile in PA alone (mean MA percentile = 25), another 19 fell below the 25th percentile in MA alone (mean PA percentile = 30) and the remaining 17 were below the 25th percentile on both measures (PA = 16, MA = 14). These data demonstrate that our PA + MA-only subgroup does not consist of children with a purely PA-deficit profile but intact MA, nor a purely MA-deficit subgroup with intact PA, but children who were impaired on both PA and MA to varying degrees.

2 Limiting the control group to the 35th-65th percentile range was designed to ensure that the group represents the average or normative performance of our cohort. Removing the 65th percentile cap would result in a control/comparison group with above-average performance artificially inflating differences between this control group and the disabled subgroups. This 35–65 range also ensured that the range of scores in both disabled and control groups (SDs and SEs) was similar which is statistically preferable.

3 In our sample, many children have not mastered all the color and object names. The errors made by this subsample (*n* = 157) often involved skipping items or a whole line which reduced the recorded time significantly thereby calling into question the validity of this measure as a reflection of name retrieval speed for this subsample. To ensure that by excluding this group (*n* = 157) we were not biasing our results in some way, we reanalyzed our data (see Supplementary Analyses reported below) by comparing the two sets of results on the Kindergarten and Grade 1 measures (including versus excluding the 157 error-prone subsample).We found a very similar pattern of results.

4 Although we regard the terms *speed* and *rate* as interchangeable, the present review adopts the term *rate* to be consistent with Lovett’s (1984, 1987) pioneering work and our own previous published studies (Shany & Share ,2011, Shany et al., 2023, and Jabbour-Daniel et al., 2024).
